# DCMP: database of cancer mutant protein domains

**DOI:** 10.1093/database/baab066

**Published:** 2021-11-13

**Authors:** Isaac Arnold Emerson, Kiran Kumar Chitluri

**Affiliations:** Bioinformatics Programming Lab, Department of Biotechnology, School of Bio Sciences and Technology, Vellore Institute of Technology, Vellore, TN 632 014, India; Bioinformatics Programming Lab, Department of Biotechnology, School of Bio Sciences and Technology, Vellore Institute of Technology, Vellore, TN 632 014, India

## Abstract

Protein domains are functional and structural units of proteins. They are responsible for a particular function that contributes to protein’s overall role. Because of this essential role, the majority of the genetic variants occur in the domains. In this study, the somatic mutations across 21 cancer types were mapped to the individual protein domains. To map the mutations to the domains, we employed the whole human proteome to predict the domains in each protein sequence and recognized about 149 668 domains. A novel Perl-API program was developed to convert the protein domain positions into genomic positions, and users can freely access them through GitHub. We determined the distribution of protein domains across 23 chromosomes with the help of these genomic positions. Interestingly, chromosome 19 has more number of protein domains in comparison with other chromosomes. Then, we mapped the cancer mutations to all the protein domains. Around 46–65% of mutations were mapped to their corresponding protein domains, and significantly mutated domains for all the cancer types were determined using the local false discovery ratio (locfdr). The chromosome positions for all the protein domains can be verified using the cross-reference ensemble database.

**Database URL**: https://dcmp.vit.ac.in/

**Key Points**

- DCMP is a web-based resource for protein domains, providing chromosome positions and cancer mutation counts.
- DCMP provides the protein domain distribution across 23 chromosomes.
- DCMP allows the user to explore significantly mutated domains across the 21 cancer types.

## Introduction

Cancers are triggered by collective changes in genetic and non-genetic materials, which are induced by environmental factors that elicit inappropriate activation or inactivation of specific genes (1). It started by way of disrupting the pathways of cellular proliferation as well as differentiation leading to neoplastic transformations or abnormal cell growth (2). It is a large family of diseases that can invade or spread to other parts of the body. Analyses of well-studied cancers, such as colorectal cancer and retinoblastoma, have suggested that only three or fewer mutations are sufficient for cancer initiation (3–5). Most researchers have carried out detailed studies that focus on how to stop this deadly disease in its tracks. One such study includes the application of genomics and proteomics in cancer biology, which holds great potential for identifying the mechanisms that lead to malignancy and the development of therapeutic strategies (6). Several cancer genomes were sequenced and documented thousands of DNA mutations and other genomic alterations (7–9). Efforts were made by the team of The Cancer Genome Atlas, the International Cancer Genome Consortium and Catalogue of Somatic Mutations in Cancer (COSMIC) (10–12). In recent years, mutational landscapes of several cancer types have been revealed. However, the extracting process of knowledge from immense sequence resources has just begun. Each cancer can contain thousands of somatic mutations that exemplify challenges to therapy and provide a basic understanding of the cancer disease.

Therefore, genomic sequence, with the chromosomal mapping data, has dramatically enhanced the ability to isolate specific genes involved in heritable cancers, such as those responsible for predisposition to breast cancer, *BRCA1* and *BRCA2* (13, 14). These are considered as potential mutation driver genes (15, 16), and also, few enzymes like histone deacetylases were identified as potential therapeutic targets (17). Targeted therapy is a newer cancer treatment that targets proteins that control how cancer cells grow, divide, and spread (18, 19), like kinases are mainly focused on these systems, either as downstream regulators in signaling pathways or as receptor molecules. A few best examples for these studies are human epidermal growth factor receptors such as *EGFR* and *HER2* (20). Since genomic profiling for all kinds of tumor has been increased eventually, the overexpression of *HER2* was identified in several tumor types, including cervical (2.2%), bladder (3.6%), salivary (3.9%), vaginal (3.6%), endometrial (3.4%) and colorectal cancers (1.3%). Similarly, 6.02% of altered *EGFR* is observed in several cancer types, including lung, breast and colon. Hence, molecule inhibitors and therapeutic drugs are being developed for *HER2* and *EGFR* with more excellent selectivity, specific to *HER2* and *EGFR* mutations (21–23).

Currently, vast data of cancer genome sequences increase with the number of tumor samples, where the prediction of driver mutations in these genomes reflects false positive rate data (24, 25). Hence, determining the effects of mutations on the structure and function of the protein remains challenging (26). Recent computational structural studies have revealed that this gene-based approach usually does not consider the position of the mutation within the gene or provides the functional context of the position of the mutation. Computational structural studies have explored mutational effects on specific regions of a protein (e.g. the binding site) (27–29). In this study, the somatic mutations of 21 different cancers were mapped to the individual protein domains to identify the significantly mutated domains (SMDs) across the cancer types. For mapping mutations, the protein domains were predicted from the human proteome, and the domain positions were converted into their nucleotide or chromosomal location. Thus, turning the peptide into a nucleotide position offered a reliable method of mapping mutations to protein domains. The top 10 significant protein domains were determined using the local false discovery ratio. The users can access the protein domain position in the chromosome with the help of a developed database.

## Materials and methods

### Human protein sequences

The human protein sequences were retrieved from Ensembl using genome assembly GRCh38.p13 (Genome Reference Consortium Human Build 38), INSDC Assembly GCA_000001405.28, December 2013 (30). The protein domains from each protein sequence were predicted using the Pfam scan tool, and we considered the domains with an e-value ≤0.01 (31).

### Prediction of protein domains from the human proteome

The homo sapiens proteome containing 109 095 sequences was obtained from the Ensembl database using genome assembly GRCh38. The PfamScan search tool is locally installed, incorporating HMMER and BLAST to search against Pfam domain libraries. The individual protein sequence of the target species was searched against Pfam libraries, and the total estimate of 169 745 protein domains was predicted. We considered 149 668 domain hits with an e-value of ≤0.01, and Table 1 represents the example output from the Pfamscan program. The PfamScan program searches a set of protein sequences in FASTA format against Pfam’s library of HMMs, and it requires the standard Perl library modules and the HMMER programs (31, 32). The following steps are necessary to install and run the PfamScan program.

**Table 1.** Illustration of predicted domains from the Pfamscan tool with an e-value ≤0.01. Each line contains the following information: 1—seq id, 2—alignment start, 3—alignment end, 4—envelope start, 5—envelope end, 6—hmm acc, 7—hmm name, 8—type, 9—hmm start, 10—hmm end, 11—hmm length, 12-bit score, 13—e-value, 14—significance and 15—clan

1	2	3	4	5	6	7	8	9	10	11	12	13	14	15
ENST00000615270.1	128	181	128	222	PF13927.1	Ig_3	Domain	1	36	75	17.7	0.0037	1	CL0011
ENST00000616914.1	128	181	128	222	PF13927.1	Ig_3	Domain	1	36	75	17.7	0.0037	1	CL0011
ENST00000615996.1	128	155	128	224	PF13927.1	Ig_3	Domain	1	28	75	16.3	0.01	1	CL0011
ENST00000611873.1	128	181	128	222	PF13927.1	Ig_3	Domain	1	36	75	17.7	0.0037	1	CL0011
ENST00000339924.12	128	181	128	222	PF13927.1	Ig_3	Domain	1	36	75	17.7	0.0037	1	CL0011
ENST00000391729.1	128	181	128	211	PF13927.1	Ig_3	Domain	1	36	75	17.8	0.0034	1	CL0011
ENST00000621713.1	128	181	128	222	PF13927.1	Ig_3	Domain	1	36	75	17.7	0.0037	1	CL0011
ENST00000610808.1	128	181	128	222	PF13927.1	Ig_3	Domain	1	36	75	17.7	0.0037	1	CL0011

To install the PfamScan program:

1. First, download the tarball ‘PfamScan.tar.gz’ and unpack the script using ‘tar zxvf PfamScan.tar.gz’ command. The standalone Perl script ‘pfam_scan.pl’ is obtained from http://ftp.ebi.ac.uk/pub/databases/Pfam/.
2. Second, compile the HMMER3 source code using the tarball of the HMMER3 beta 3 release from the HMMER site http://hmmer.org/download.html and add HMMER3 binaries to your path.
3. Install non-standard Perl dependencies, the Moose framework and Bioperl 1.4 via CPAN.
4. Finally, add the Pfam Modules to your PERL5LIB using the ‘export PERL5LIB=/path/to/pfam_scanDir:$PERL5LIB’ bash command.

To run searches using ‘pfam_scan.pl’:

1. Download Pfam data files, Pfam-A.hmm, Pfam-A.hmm.dat, Pfam-B.hmm, Pfam-B.hmm.dat and active_site.dat from the Pfam FTP site http://ftp.ebi.ac.uk/pub/databases/Pfam/current_release/.
2. Generate the binary files for Pfam-A.hmm and Pfam-B.hmm by running the following commands: hmmpress Pfam-A.hmm and hmmpress Pfam-B.hmm.
3. Input the protein sequences in a FASTA-format file containing your query sequence(s).
4. Run the program using ‘pfam_scan.pl -fasta <fasta_file> -dir <directory location of Pfam files>’.

### Cancer mutations from the COMIC database

The COSMIC database was used to download the mutations for 21 different cancers, using the GRCh38 genome version, as shown in Table 2. The mutations were obtained under the COSMIC Complete Mutation Data (Targeted Screens) that contains the tab-separated table of the complete, curated COSMIC dataset in January 2020 (33). It is the most comprehensive resource for exploring the impact of somatic mutations in human cancer. The mutation types, such as nonsense, missense, coding silent and complex, which involve multiple insertions, deletions and substitutions, were included. Intronic and unknown mutations were excluded from the mutation dataset because those mutations occur outside the coding domains and mutations with no detailed information.

**Table 2.** Cancer primary types and their mutation counts

S. no.	Cancer primary site	No of mutations
1	Adrenal	10 868
2	Biliary	69 570
3	Bone	34 139
4	Brain	129 130
5	Breast	285 712
6	Cervix	55 642
7	Endometrium	282 168
8	Eye	2085
9	Kidney	112 577
10	Large interstine	1 039 252
11	Liver	409 309
12	Lung	670 483
13	Esophagus	214 073
14	Ovary	70 245
15	Pancreas	126 512
16	Prostate	151 061
17	Skin	921 194
18	Stomach	276 849
19	Testis	1179
20	Thyroid	258 180
21	Urinary	235 499

### Mapping cancer mutation to protein domains

**Figure 1.**
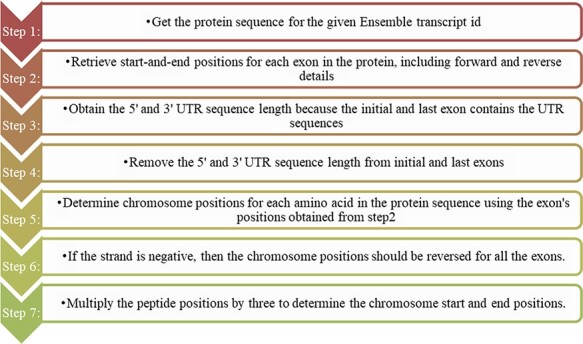
Steps for converting protein domain position to genome position.

The domains predicted from the protein sequence are reported in peptide position, whereas the cancer mutations are depicted in genomic locations. Before mapping the cancer mutations to their corresponding protein domains, we should change either the mutation or domain positions. In this study, we choose to change the domain positions to their genomic positions. A Perl program was written using the ensemble Perl API module to convert the protein domain positions into genomic positions, and the steps followed are shown in Figure 1. We did not consider the intron positions and extracted only the exon positions as this code for amino acids. In addition to the exon position, we retrieved the 3ʹ and 5ʹ UTR positions as they were present in the initial exon and last exon. Combining all the exon positions results in a complete coding sequence. Finally, the genomic coding positions are divided by 3 to represent the actual amino acid count. The users can freely access the Perl-API program from the GitHub link https://github.com/iarnoldemerson/Protein-to-genome-position.git, and supplementary file 1 provides the program instruction.

**Figure 2.**
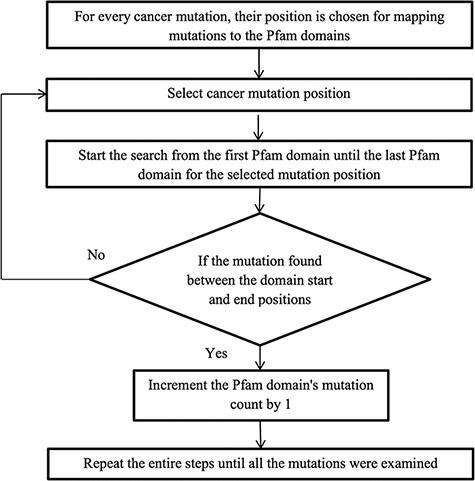
Flowchart for mapping mutations to the protein domains.

After converting the domain position into genomic position using the Perl API program, cancer mutations are now ready to map with their protein domains. Figure 2 illustrates the methodology for mapping the mutation to the protein domains. Every mutation is searched through all the Pfam domains. If the mutation position is detected between the domain start and end, then the mutation count is increased by 1, else choose the next mutation. Some mutations do not map to any Pfam domains, and this is because the mutation is not positioned in the protein domain locations.

### Calculation of normalized mutation frequency and SMDs

**Figure 3.**
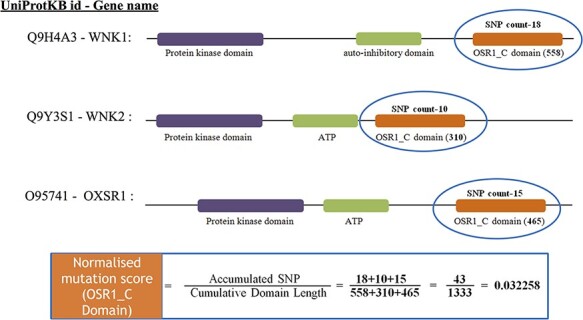
The estimate of normalized mutation count for the OSR1_C domain.

After mapping all the cancer mutations, the mutation count for each Pfam domain needs to be normalized. In this study, we normalized the mutation counts by utilizing the cumulative length of all occurrences of the Pfam domain within the cancer set. Figure 3 depicts an illustration of normalizing the OSR1_C domain, and it is located in three genes, namely, *WNK1*, *WNK2* and *OXSR1*. The accumulated SNP signifies the sum of mutations that occurred in the OSR1_C domains, whereas the cumulative domain length is accomplished by summing their domain length in all those three genes.

To determine the SMDs, we adapted the method to estimate the local false discovery rate in microarray experiments by Efron *et al.* The relative frequency is utilized as the success probability (*p*). Then, it was normalized using the Bernoulli distribution signal to noise ratio, which results in the normalized score, *z*, as follows:

$$\begin{equation*}Z = p/sqrt\left( {p\left( {1 - p} \right)} \right)\end{equation*}$$

The null distribution is estimated using the ‘locfdr’ package from R and employed these statistics to identify statistically significant domains with a local false discovery rate of <0.1. False Discovery Rate (FDR) controls the number of false positives that result in a significant result, and it has a greater ability to find truly significant results. For example, an FDR of 0.1 implies that 10% of significant tests will result in false positives. In a gene expression study, when the FDR was fixed at 0.1, seven genes with a significant difference were found. However, the number of significant differences decreases to 1, using a more stringent FDR of 0.05. Furthermore, it has been shown that the number of false positives recovered is considerably higher than the number expected (34). Thus we have chosen the FDR of 0.1 to reduce the false positive in the SMDs. We created a heat map representation of the hierarchical clustering of SMDs in different cancers using the ‘heatmap’ R package based on the ‘locfdr’ values.

## Results and discussion

### Conversion of protein to genomic positions

**Figure 4.**
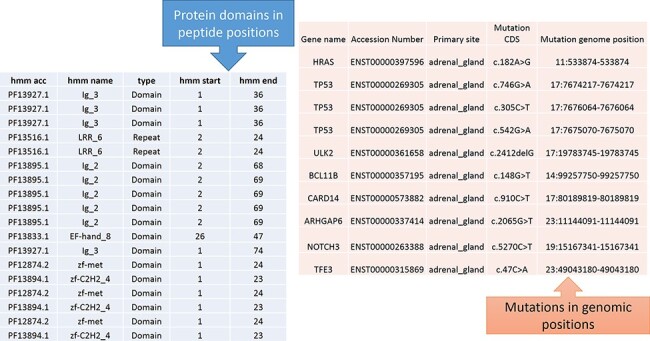
Predicted protein domains with peptide start and end (blue table) and cancer mutations are represented in genomic positions for the adrenal gland (pink table).

From Figure 1, the protein domains were predicted with the peptide positions, whereas the cancer mutations were reported with genomic or chromosome positions. To accomplish the mapping of the cancer mutations to the protein domain, either the peptide positions or the chromosome positions need to be converted. The most efficient method is to convert peptide positions to their corresponding chromosome positions. Thus, it creates a more straightforward way to map all the cancer mutations to the protein domains. The steps required for converting peptides to chromosome positions are described in the ‘Materials and methods’ section (Figure 4).

**Figure 5.**
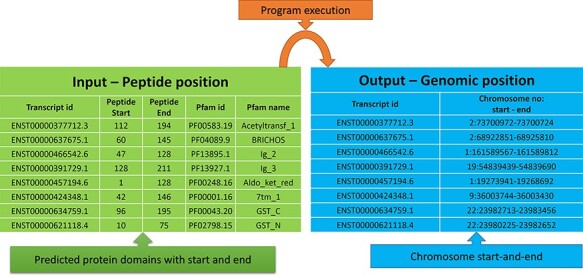
Input and output features for the peptide to genome program execution. The green table indicates the peptide positions for each transcript id, and the program’s output provides the genomic locations as depicted in the blue table.

The peptide to genome conversion program takes peptide start and end as an input (Figure 5—green table), and it provides their corresponding chromosome positions as output (Figure 5—blue table). The program output can be validated using the transcript id in the Ensemble database. For example, the first transcript id ENST00000377712.3 in Figure 5, the Aetyltransf_1 domain, starts from 112 to 194, containing 83 amino acids. Since each amino acid contains three nucleotides, it requires 249 bases. The result shows that the Pfam domain resides in the second chromosome, and it starts from 73 700 972 to 73 700 724 (negative strand). Thus, the total length is equal to 249 bases, which codes for 83 amino acids. This equality is not the case in many chromosome positions. This transcript contains only one exon without introns, where the chromosome length is precisely equal to the peptide length (i.e. 249/3 nucleotides = 83 amino acids).

**Figure 6.**
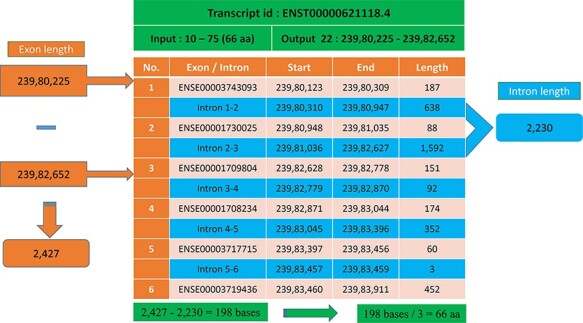
Transcript with multiple exons and introns. The Pfam domain GST_N length is 66aa, where the actual bases are obtained by subtracting the intron length (blue) from the genomic positions (orange).

In most cases, the transcript will have multiple exons and introns, and the protein domain starts and ends in different exons. One such example is the last transcript id ENST00000621118.4 (Figure 5), in which the GST_N domain begins from 10 to 75, comprising 66 amino acids and requires 198 (66 × 3) nucleotide bases. Whereas this GST_N domain is present in the 22nd chromosome between 23 980 225 and 23 982 652 (forward strand), the total length is equal to 2427 bases. Instead of 198 bases, the program provides 2427 bases; this is because the transcript contains six exons and five introns. The initial chromosome position 23 980 225 resides in the first exon, and the last chromosome, position 23 982 652, ends in the third exon. Between these two exons, there are two introns of size 638, and 1592 bases are located, which equals 2230 bases. The program output is 2427 bases, and the intron length is 2230 bases. Thus, if we subtract the intron length from the total length (2427 − 2230 = 198 bases), the actual 198 bases that code 66 amino acids are remaining, as shown in Figure 6.

### Protein domains in human chromosomes

**Figure 7.**
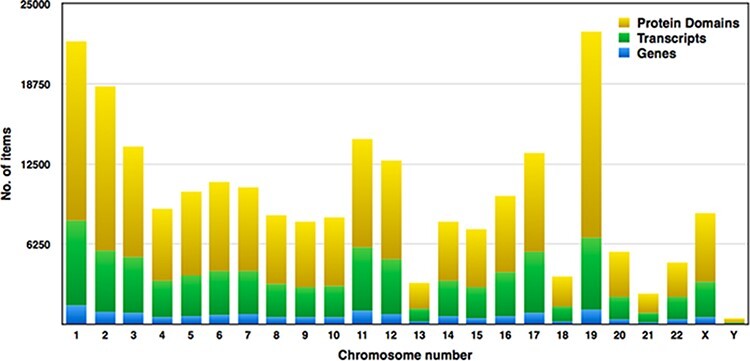
Distribution of genes, transcripts and protein domains within human chromosomes.

Pfam domains with ≤0.01 were selected for higher accuracy, and subsequently, we examined around 149 668 domains from the human proteome. Each chromosome contains hundreds to thousands of genes, which carry the instructions for making proteins. Each of the estimated 30 000 genes in the human genome makes an average of three proteins. A single gene can produce multiple different RNAs, i.e. transcripts. The actual transcript observed will depend on the tissue, developmental time point, environmental factors, etc. The number of coding genes and protein-coding transcripts in each chromosome was determined and compared with the number of protein domains across 23 chromosomes, as shown in Figure 7. In our study, the estimated number of unique genes is around 15 096, and these genes account for 73 311 transcripts, and thus, the average number of transcripts per gene is 4.85%. Figure 7 represents the distribution of Pfam domains across 23 chromosomes. Interestingly, the 19th chromosome had more Pfam domains, and it was estimated as 16 091; on the contrary, chromosome y had fewer domains, which is 314. The chromosome positions of each protein domain can be verified using the cross-reference ensemble database; see step 1 with the help manual for more details (Supplementary file 2). The user can obtain the chromosome details for any given protein domain, including chr_no., chr_start and chr_end position, and strand (positive or negative) details, under the ‘Domain genomic positions’ menu in the database.

### Mapping mutations to individual protein domains

**Figure 8.**
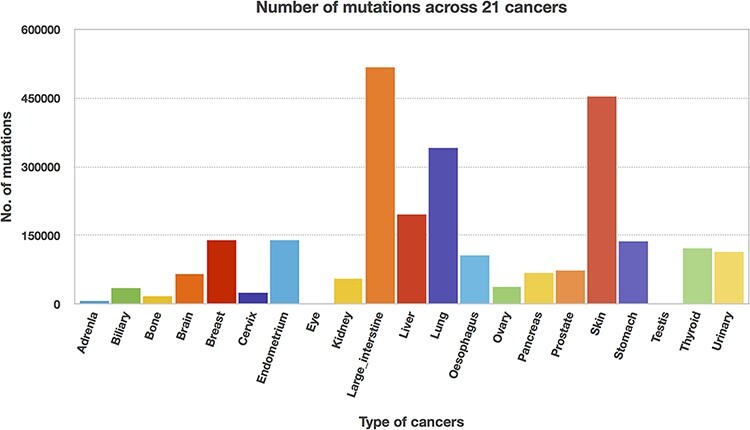
Mutation counts across 21 cancer types.

We utilized the developed Perl API program to transform all the Pfam domain positions into their chromosome positions. Thus, the mutation and domain positions became precisely equivalent in their locations (i.e. chromosome position). The next step is to map the mutations into each protein domain, and this step requires more computation time since the mutation position is compared with all the domain positions. Mapping of mutations was carried out for all 21 cancer types, and Table 3 represents the percentage of mutations mapped to the protein domains. The percentages of mutations range from 46 to 65, suggesting that the non-mapped mutations are not in the protein domain location. In addition, mutated domains were also calculated and depicted in Table 3. After the mutations were mapped to individual protein domains, we calculated the number of mutations in each cancer type. Interestingly, we found that the “large intestine” cancer acquired more mutations for 518 025, as shown in Figure 8. 

**Table 3.** Percentage of mutations mapped to the protein domains

Cancer type	COSMIC mutation data	No. of mutated protein domains	No. of mutations mapped to domains	Percentage
Adrenal	10 868	1322	6020	55.39
Biliary	69 570	3377	34 141	49.07
Bone	34 139	2594	16 957	49.67
Brain	129 130	3791	66 615	51.58
Breast	285 712	4901	140 430	49.15
Cervix	55 642	3280	26 009	46.74
Endometrium	282 168	4952	139 785	49.53
Eye	2085	209	1376	65.99
Kidney	112 577	4117	56 746	50.40
Large interstine	1 039 252	5327	518 025	49.84
Liver	409 309	5093	195 589	47.78
Lung	670 483	5190	340 620	50.80
Esophagus	214 073	4423	106 436	49.71
Ovary	70 245	3402	36 772	52.34
Pancreas	126 512	3771	67 563	53.40
Prostate	151 061	4286	73 305	48.52
Skin	921 194	5217	453 763	49.25
Stomach	276 849	4822	137 911	49.81
Testis	1179	198	668	56.65
Thyroid	258 180	4195	122 263	47.35
Urinary	235 499	4714	114 324	48.54

### Significantly mutated domains

**Figure 9.**
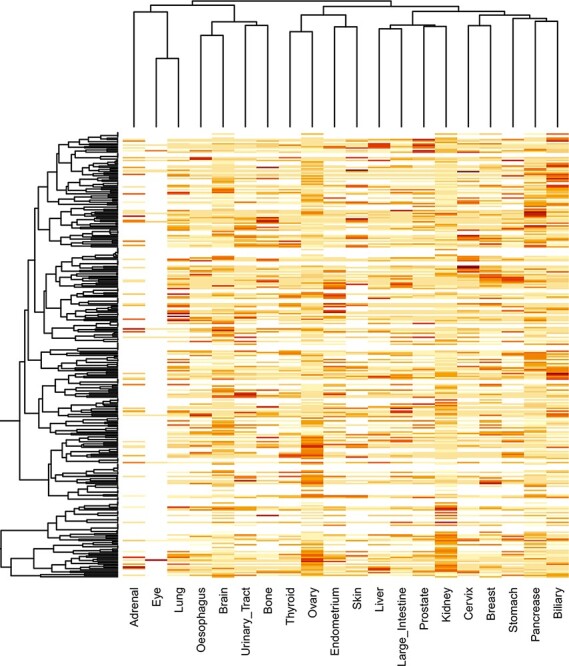
Clustering of significantly mutated domains (SMDs) across different cancer types. The heatmap reveals the importance of cancer-specific SMDs in various cancers. The sidebars in the same color represent the domain instances belonging to the specific cancer type.

The locfdr was used to determine the statistically significant domains for all the cancer types. The top 10 protein domains in each cancer type are shown in Supplementary file 3. The total number of SMDs across 21 cancer types is 3431 out of 79 181, accounting for ∼4.33% of protein domains. The list of SMDs (3431) and the list of mutated domains (79 181) are provided in Supplementary files 4 and 5, respectively. In addition, these mutated domains for 21 cancers can also be obtained through our developed database under the ‘Mutated Domains’ menu (http://dcmp.vit.ac.in/mutated_domains/). The distribution of SMDs varies across cancer types, as depicted using heatmap in Figure 9. Among cancer-specific SMDs, most were only significantly mutated in a single cancer type. Thus, each column represents cancer, and the same color indicates the SMDs belong to the particular cancer type. Moreover, the P53 was the only domain observed in the significantly mutated domain of the “testis” cancer type, and we excluded it in the heatmap.

Interestingly, the p53 protein domain has been found in the top 10 list of all cancer types. The *TP53* gene is a gene that is mutated in many cancers, and it is the most common gene mutation found in cancer cells. A tumor-suppressor gene, *TP53*, codes for a protein that inhibits the development and growth of tumors. Since over 50% of human cancers carry loss of function mutations in the *p53* gene, *p53* has been considered one of the classical type tumor suppressors. There are three protein domains, namely, PI3Ka, Nebulin and zf-H2C2_2, which occur in >10 cancer subtypes. PI3Ka is believed to be one of the significant therapeutic targets for cancer treatment (35). Hyperactivity of PI3K signaling is significantly associated with human tumor progression and invasive potential of cancer cells. *NEBL* (nebulette) gene is located on chromosome 10p12.31 and encodes the nebulin-like protein, and studies indicate the role of *NEBL* as an oncogene and tumor suppressor in cancer (36). The ZF domains are significant determinants of human regulatory networks, as they are contained in nearly half of human transcription factors. Studies establish that mutation in *ZF* genes is expressed at levels comparable to other cancer-relevant genes (37).

### Access to the database

The database facilitates users to explore mutated protein domains for different cancer types, and it consists of the following three primary menus: domain genomic positions, SMDs and mutated domains. The initial one represents the chromosome positions for any given protein domain with references to the ensemble. The second menu displays the top 10 SMDs with references to the Pfam database. The last menu provides a complete list of mutated protein domains for any given cancer type. The front-end was designed using PHP scripting language with MySQL as the database, and the interactive graphs were plotted using CanvasJS. The DCMP database can be reached through the weblink http://dcmp.vit.ac.in/.

## Conclusions

Mutation in the protein contributes specific information than a normal protein. It can cause cells to multiply uncontrollably and become cancerous. Identification of mutated proteins in the cell is an essential part of developing novel therapeutic targets. The whole human proteome was used to determine the mutated domains in 21 different cancer types. Somatic mutations were mapped to the protein domains, and the SMDs were selected using statistical methods. Users can visualize the genomic positions of any protein domain and the list of mutated domains in 21 cancer types using the DCMP database.

## Supplementary Material

baab066_Supp
