# Supplementary material for: DCMP: database of cancer mutant protein domains
Source: Database (Oxford). 2021 Nov 13;2021:baab066. doi: 10.1093/database/baab066 (PMC8607521; doi:10.1093/database/baab066)
Supplement: baab066_Supp [file baab066_supp.zip › supplementary_file1.pdf]

Instructions for executing the Perl-API program

Download the Perl program from the below GitHub link:
https://github.com/iarnoldemerson/Protein-to-genome-position.git

# Protein-to-genome-position
Perl program to convert the protein position into genomic position

1) Required Perl modules

```
use Bio::EnsEMBL::DBSQL::DBAdaptor;
use Bio::EnsEMBL::Registry;
use Bio::EnsEMBL::Translation;
use Bio::EnsEMBL::Transcript;
```

2) Input format

Create a text file "input.txt" as file name and save the input in the below format that contains five column details: Ensemble_id, domain_start, domain_end, Pfam_id, Pfam_name

ENST00000390396.1	20	115	PF07686.12	V-set
ENST00000390400.2	20	114	PF07686.12	V-set
ENST00000621184.1	20	115	PF07686.12	V-set
ENST00000390372.3	20	114	PF07686.12	V-set
ENST00000390369.2	20	115	PF07686.12	V-set

3) How to run the program

Type the below command at the command or terminal prompt and place the input.txt file in the same folder where the Perl program is saved

For Windows: C:\> perl protein_to_genome.pl
For Linux: $ perl protein_to_genome.pl

4) Output format

The output of the program is stored in the “output.txt” file with two column details: Ensemble_id, Chr_no:start-end

ENST00000390396.1	7:142646178-142646465
ENST00000390400.2	7:142720874-142721158
ENST00000621184.1	7:142581138-142581425
ENST00000390372.3	7:142482734-142483018
ENST00000390369.2	7:142455346-142455633
