# Supplementary material for: DCMP: database of cancer mutant protein domains
Source: Database (Oxford). 2021 Nov 13;2021:baab066. doi: 10.1093/database/baab066 (PMC8607521; doi:10.1093/database/baab066)

Help Manual for DCMP: Database of Cancer Mutant Protein Domains

1. “Domain Genomic Positions”

2. “Significantly Mutated Domains”

3. “Mutated Domains”

1. “Domain Genomic Positions”

How to verify the protein domain chromosome positions

a) Under the “Domain genomic positions” menu, type “AMP-Binding” in the search box. The below table will appear after you click the “search” button. To verify the chromosome position, click any ensemble id where the AMP-domain is located. The red circle shows the position where the domain is located.

Ensemble_ID	Chromosome	Start	End	Strand	Domain_length
ENST00000513376.5	4	56378437	56354074	-	324
ENST00000205214.10	4	56384281	56354074	-	444
ENST00000514745.5	4	56384281	56371640	-	113
ENST00000502617.1	4	56384281	56354074	-	444
ENST00000602986.5	4	56378353	56354074	-	296
ENST00000451613.5	4	56384281	56354074	-	444
ENST00000289416.9	16	20770245	20792125	+	414

b) Click the “Show transcript table” button to display all the transcript under the location

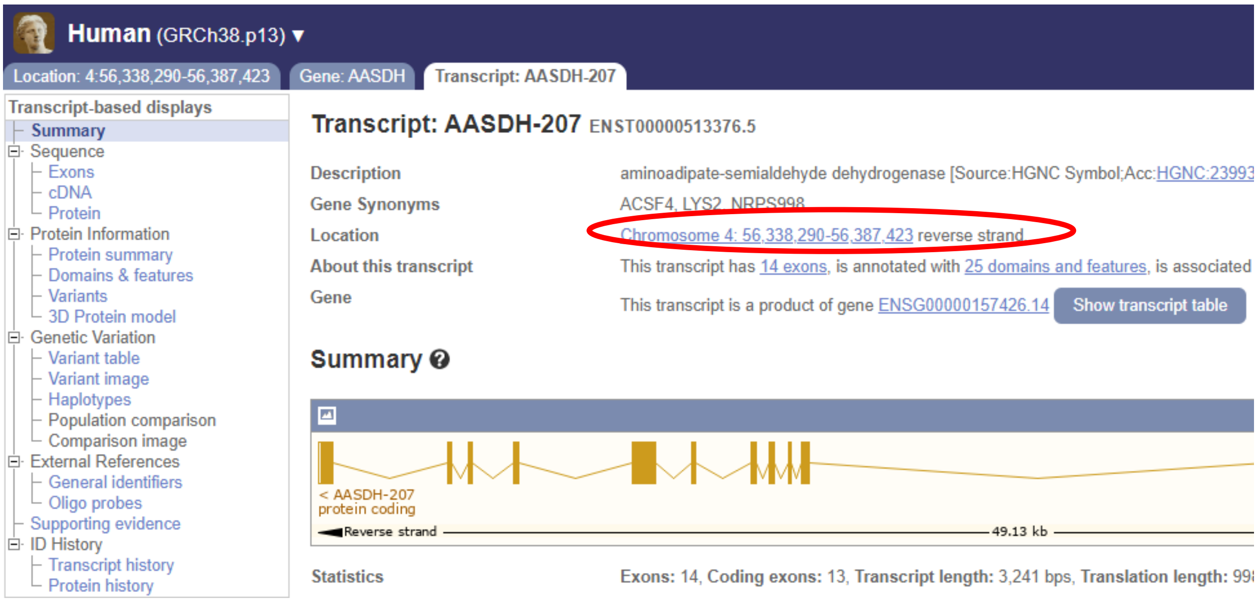

c) To display the Pfam domain, click the “Uniprot ID” and it will display the protein sequence information

Gene

This transcript is a product of gene ENSG00000157426.14 Hide transcript table

Show/hide columns (1 hidden)

Name	Transcript ID	bp	Protein	Biotype	CCDS	UniProt Match	RefSeq Match
AASDH-201	ENST00000205214.11	3581	1098aa	Protein coding	CCDS3504	Q4L235-1	NM_181806.4
AASDH-210	ENST00000602986.5	3480	945aa	Protein coding	CCDS75126	R4GNB1	-
AASDH-207	ENST00000513376.5	3241	998aa	Protein coding	CCDS68705	Q4L235-2	-
AASDH-202	ENST00000451613.5	3182	857aa	Protein coding	CCDS68706	Q4L235-4	-
AASDH-203	ENST00000502617.1	3004	841aa	Protein coding	CCDS75127	Q4L235-3	-
AASDH-208	ENST00000514745.5	2959	120aa	Nonsense mediated decay	-	D6RJA2	-
AASDH-204	ENST00000503808.5	2949	86aa	Nonsense mediated decay	-	E9PH98	-
AASDH-206	ENST00000510762.1	538	No protein	Processed transcript	-	-	-
AASDH-205	ENST00000510012.1	1222	No protein	Retained intron	-	-	-
AASDH-209	ENST00000514796.1	404	No protein	Retained intron	-	-	-

d) From the UniProt entry “Q4L235-1”, click “View protein in Pfam” under the “Family and domain databases”

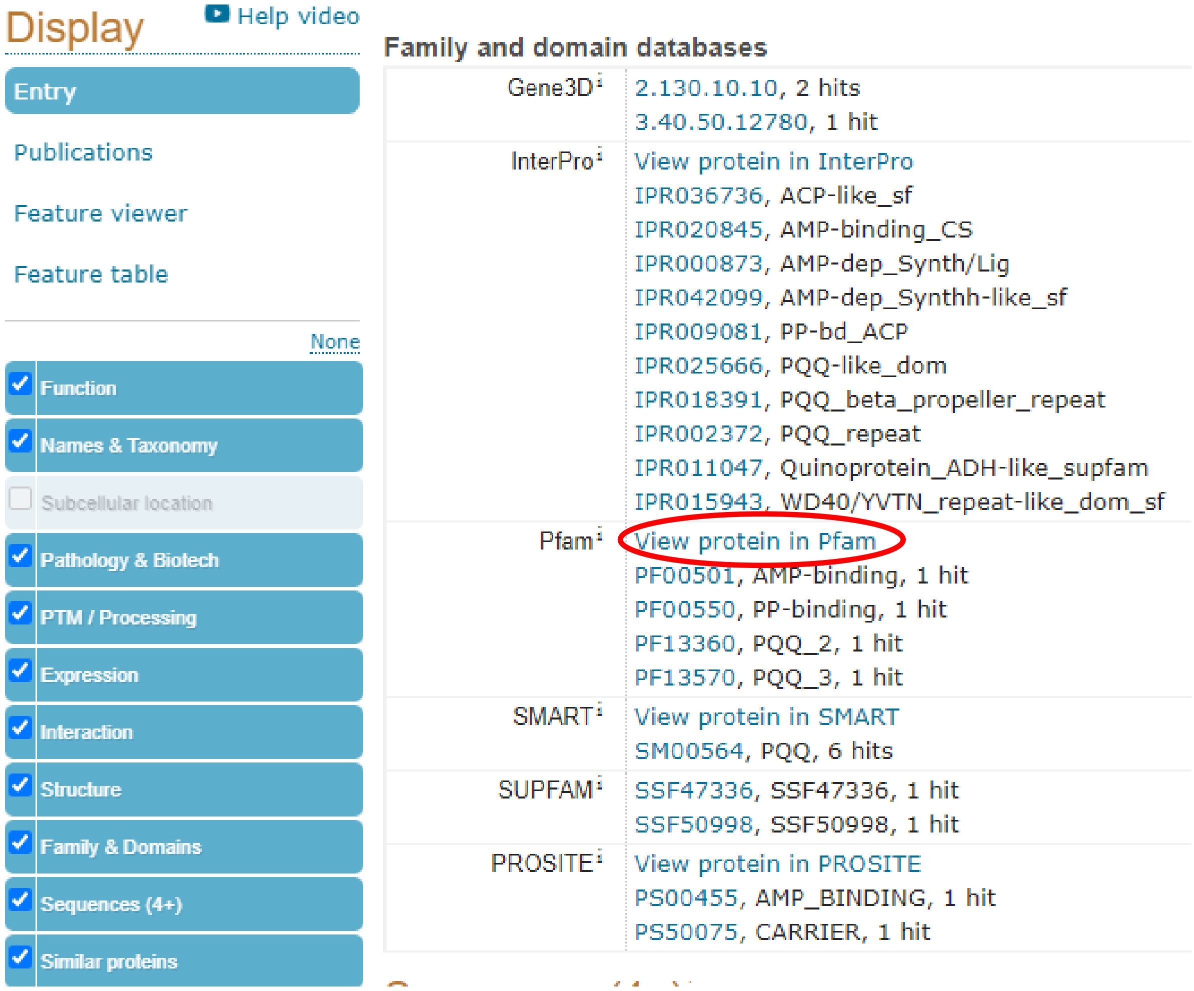

e) The user can view the “AMP-binding” domain architecture and the domain positions in the Pfam database summary

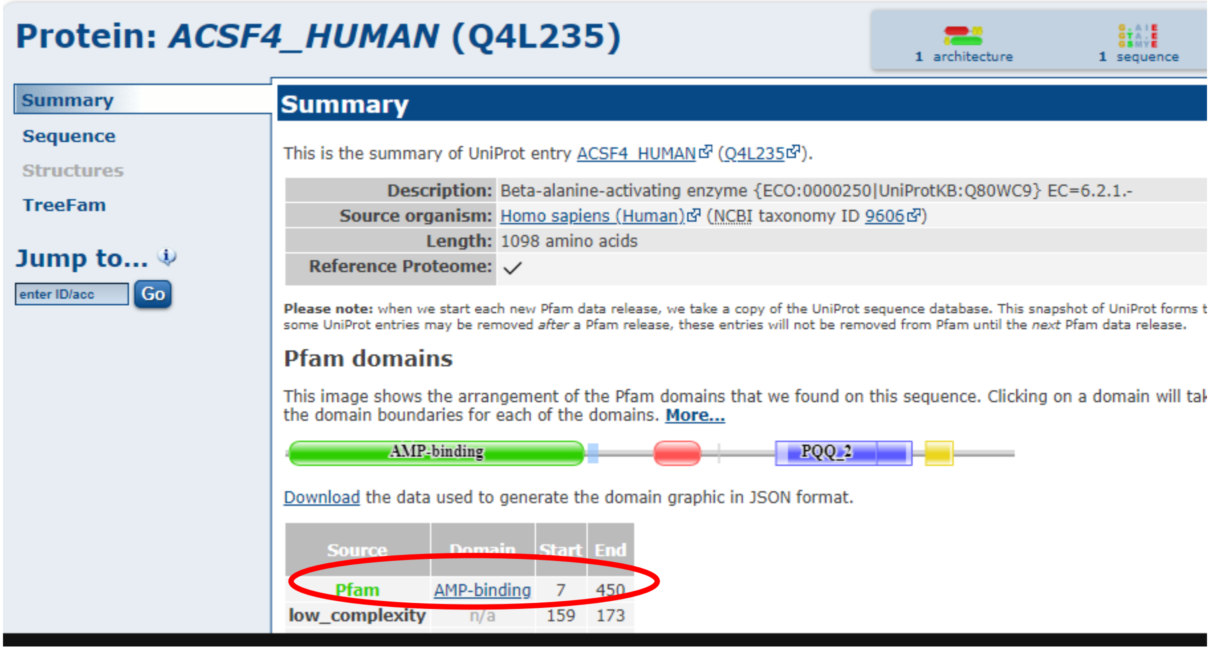

2. “Significantly Mutated Domains”

a) Type "biliary" in the search box under the "Significantly mutated domains" and click the "search" button. The table will display the top 10 significantly mutated domains with the pie chart on the left side. The table is sorted based on the "locfdr" values.

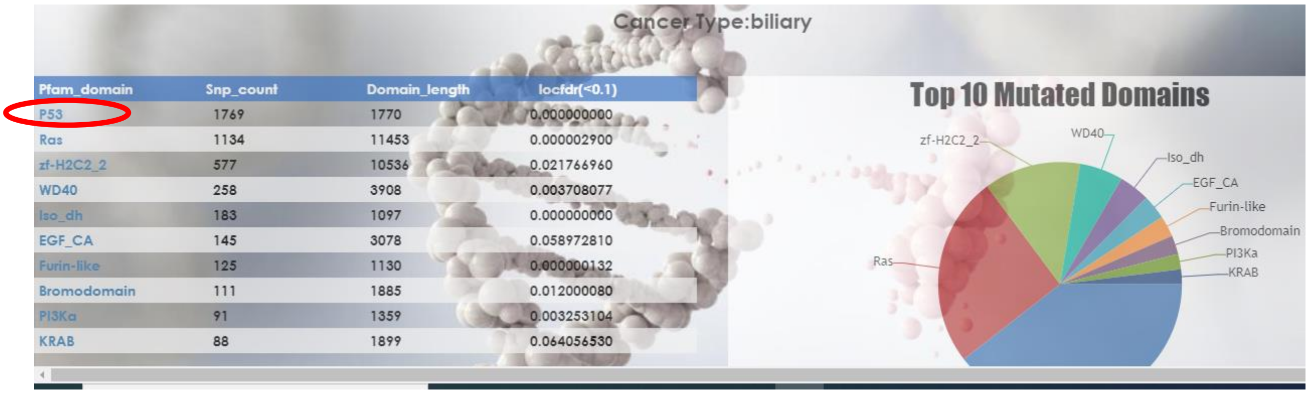
Cancer Type:biliary

Pfam_domain	Snp_count	Domain_length	locfdr(<0.1)
P53	1769	1770	0.000000000
Ras	1134	11453	0.000002900
zf-H2C2_2	577	10536	0.021766960
WD40	258	3908	0.003708077
Iso_dh	183	1097	0.000000000
EGF_CA	145	3078	0.058972810
Furin-like	125	1130	0.000000132
Bromodomain	111	1885	0.012000080
PI3Ka	91	1359	0.003253104
KRAB	88	1899	0.064056530

b) The Pfam domain details are cross-reference in the above table, and by clicking the domain name, it will direct to the Pfam database as shown below.

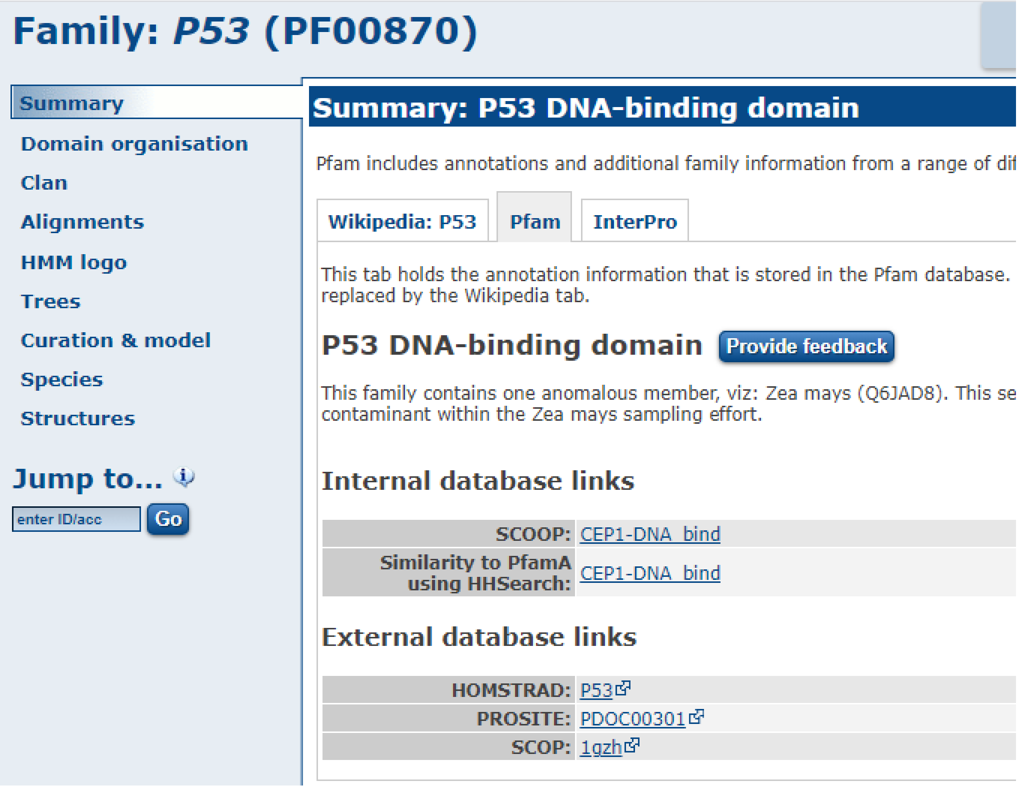
Family: *P53* (PF00870)

Summary
Domain organisation
Clan
Alignments
HMM logo
Trees
Curation & model
Species
Structures

Jump to...
enter ID/acc Go

Summary: P53 DNA-binding domain

Pfam includes annotations and additional family information from a range of dif

Wikipedia: P53 | Pfam | InterPro

This tab holds the annotation information that is stored in the Pfam database. replaced by the Wikipedia tab.

P53 DNA-binding domain Provide feedback

This family contains one anomalous member, viz: Zea mays (Q6JAD8). This se contaminant within the Zea mays sampling effort.

Internal database links

SCOOP:	CEP1-DNA_bind
Similarity to PfamA using HHSearch:	CEP1-DNA_bind

External database links

HOMSTRAD:	P53
PROSITE:	PDOC00301
SCOP:	1gzh

3. “Mutated Domains”

a) Type "biliary" in the search box under the "Mutated domains" menu and click the "search" button. The table will display all the mutated domains in particular cancer. The bar chart shows the number of mutations across 21 cancers, and the table is sorted based on the "Snp_count" values.

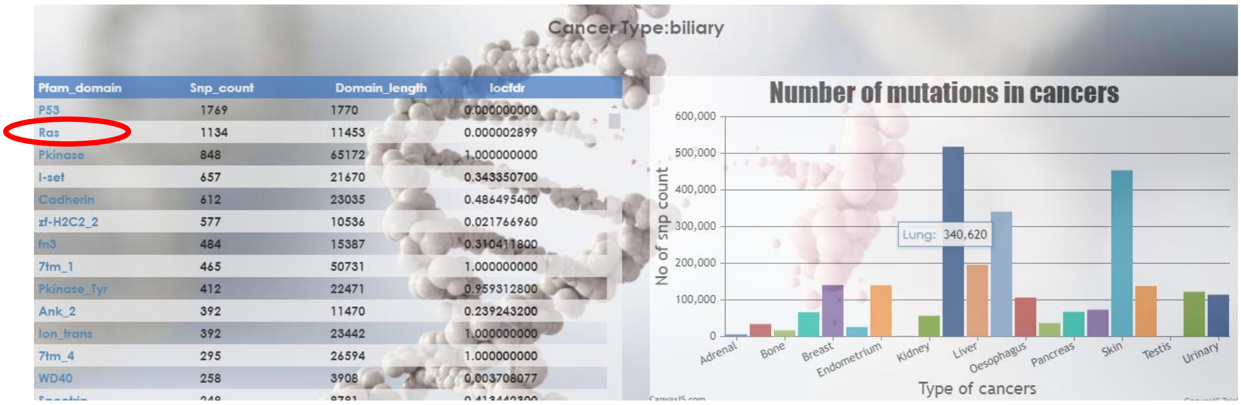

b) The Pfam domain details are cross-reference in the above table, and by clicking the domain name, it will direct to the Pfam database as shown below.

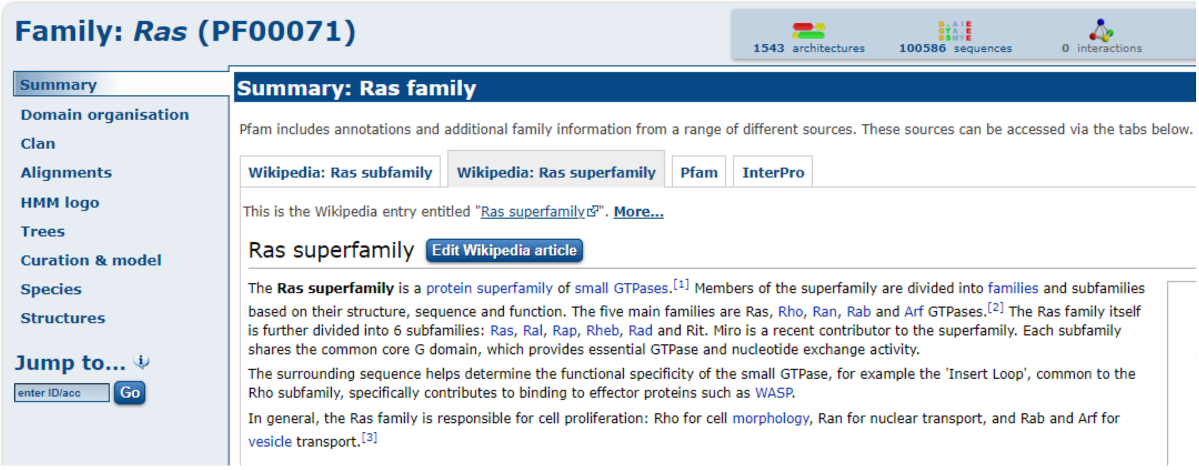

Supplement: baab066_Supp [file baab066_supp.zip › supplementary_file2.pdf]
