# Supplementary material for: DCMP: database of cancer mutant protein domains
Source: Database (Oxford). 2021 Nov 13;2021:baab066. doi: 10.1093/database/baab066 (PMC8607521; doi:10.1093/database/baab066)
Supplement: baab066_Supp [file baab066_supp.zip › supplementary_file3.pdf]

The top ten significantly mutated domains for each cancer type

Adrenal	Snp_count	Biliary	Snp_count	Bone	Snp_count	Brain	Snp_count	Breast	Snp_count
IRK	1062	P53	1769	P53	542	P53	5359	P53	10798
P53	252	Ras	1134	zf-H2C2_2	418	Iso_dh	4961	PI3Ka	1958
zf-H2C2_2	61	zf-H2C2_2	577	Iso_dh	323	zf-H2C2_2	1410	Hormone_recep	992
VHL	48	WD40	258	G-alpha	233	Furin-like	486	Furin-like	305
Cation_ATPase_N	45	Iso_dh	183	PNP_UDP_1	85	PNP_UDP_1	443	PI3K_C2	300
KRAB	22	EGF_CA	145	Pro-rich	42	DSPc	440	Runt	249
HIF-1	21	Furin-like	125	Ank	38	MIP	288	CBF_beta	232
HLH	11	Bromodomain	111	PHD	28	PTEN_C2	284	RB_A	227
RB_A	10	PI3Ka	91	RB_A	28	WD40	272	Nebulin	204
VWC	10	KRAB	88	EGF	24	MHC_I	251	zf-C2H2	202
Cervix	Snp_count	Endometrium	Snp_count	Eye	Snp_count	Kidney	Snp_count	Large_interstine	Snp_count
zf-H2C2_2	653	P53	1790	G-alpha	583	VHL	3006	Ras	22094
P53	223	DSPc	1000	Pkinase_Tyr	118	P53	1411	P53	11436
WD40	209	PTEN_C2	633	Peptidase_C12	118	zf-H2C2_2	1137	Pkinase_Tyr	8639
PI3Ka	182	PI3Ka	362	RB_A	78	Bromodomain	813	PI3Ka	1560
Histone	111	PI3K_p85B	312	P53	45	BAH	378	Nebulin	863
KRAB	86	Nebulin	308	Ras	41	WD40	304	MH2	814
Keratin_B2_2	83	DNA_pol_B_exo1	222	WD40	16	SET	220	Plectin	728
Gla	48	MIR	206	DUF3452	13	UPF0640	209	APC_crr	691
MAGE_N	46	Gla	175	DSPc	9	WT1	182	CTNNB1_binding	598
Ank	39	HEAT	169	eIF-1a	6	Homeobox	171	WTX	545
Liver	Snp_count	Lung	Snp_count	Esophagus	Snp_count	Ovary	Snp_count	Pancreas	Snp_count
P53	4452	P53	13408	P53	8370	P53	4069	Ras	8527
KRAB	1298	Pkinase_Tyr	10463	PI3Ka	277	Ras	1030	P53	5189
Nebulin	343	Ras	9502	EGF	267	Fork_head	787	zf-H2C2_2	1108
HNF-1B_C	220	Vinculin	811	Nebulin	215	zf-H2C2_2	569	Ank_2	1010
Gla	172	FAM47	483	Ank	144	PI3Ka	197	G-alpha	615
IL6Ra-bind	151	RB_A	474	Plectin	118	WD40	185	Sushi	405
DUF1220	143	PI3Ka	456	KAT11	97	TSP_1	114	WD40	304
Filaggrin	134	Ephrin_lbd	446	CLN5	82	KRAB	109	MH2	283
RB_A	134	Plectin	396	GVQW	76	EGF_CA	94	MH1	177
Spy1	125	Furin-like	342	PNP_UDP_1	74	Ribonuclease_3	84	Nebulin	165

Prostate	Snp_count	Skin	Snp_count	Stomach	Snp_count	Testis	Snp_count	Thyroid	Snp_count
P53	2706	Pkinase_Tyr	12208	P53	3921	Pkinase_Tyr	116	Pkinase_Tyr	15739
zf-H2C2_2	1389	P53	4277	Plectin	206	Ras	103	Ras	1713
Hormone_recep	669	Glyco_hydro_31	1055	Furin-like	185	P53	53	zf-H2C2_2	1628
MATH	257	Nebulin	830	PI3Ka	182	Pkinase	20	P53	1356
Fork_head	196	Ephrin_lbd	727	Nebulin	171	fn3	16	MIP	729
Keratin_B2_2	168	Na_trans_assoc	606	MIR	166	HLH	12	MHC_I	609
zf-C2H2	134	MIR	553	Recep_L_domain	152	Cadherin	9	CCDC144C	573
PTEN_C2	76	Pro-rich	507	Ank	140	PI3Ka	8	Keratin_B2_2	410
Nebulin	75	NMDAR2_C	481	ecTbetaR2	130	MH2	7	KRAB	379
RB_A	63	AIG1	476	CLN5	100	V-set	7	Pro-rich	212

Urinary	Snp_count
P53	3684
PI3Ka	534
Furin-like	368
RB_A	273
zf-C2H2	190
Recep_L_domain	186
Nebulin	133
CDI	128
Plectin	128
PNP_UDP_1	125
